# The *SLEEPER* genes: a transposase-derived angiosperm-specific gene family

**DOI:** 10.1186/1471-2229-12-192

**Published:** 2012-10-16

**Authors:** Marijn Knip, Sylvia de Pater, Paul JJ Hooykaas

**Affiliations:** 1 Department of Molecular and Developmental Genetics, Institute of Biology, Leiden University, Sylviusweg 72, 2333 BE, Leiden, The Netherlands

## Abstract

**Background:**

*DAYSLEEPER* encodes a domesticated transposase from the hAT-superfamily, which is essential for development in *Arabidopsis thaliana*. Little is known about the presence of *DAYSLEEPER* orthologs in other species, or how and when it was domesticated. We studied the presence of *DAYSLEEPER* orthologs in plants and propose a model for the domestication of the ancestral *DAYSLEEPER* gene in angiosperms.

**Results:**

Using specific BLAST searches in genomic and EST libraries, we found that *DAYSLEEPER*-like genes (hereafter called *SLEEPER* genes) are unique to angiosperms. Basal angiosperms as well as grasses (Poaceae) and dicotyledonous plants possess such putative orthologous genes, but *SLEEPER*-family genes were not found in gymnosperms, mosses and algae. Most species contain more than one *SLEEPER* gene. All *SLEEPER*s contain a C_2_H_2_ type BED-zinc finger domain and a hATC dimerization domain. We designated 3 motifs, partly overlapping the BED-zinc finger and dimerization domain, which are hallmark features in the *SLEEPER* family. Although *SLEEPER* genes are structurally conserved between species, constructs with *SLEEPER* genes from grapevine and rice did not complement the *daysleeper* phenotype in Arabidopsis, when expressed under control of the *DAYSLEEPER* promoter. However these constructs did cause a dominant phenotype when expressed in Arabidopsis. Rice plant lines with an insertion in the *RICESLEEPER*1 or 2 locus displayed phenotypic abnormalities, indicating that these genes are functional and important for normal development in rice. We suggest a model in which we hypothesize that an ancestral hAT transposase was retrocopied and stably integrated in the genome during early angiosperm evolution. Evidence is also presented for more recent retroposition events of *SLEEPER* genes, such as an event in the rice genome, which gave rise to the *RICESLEEPER*1 and 2 genes.

**Conclusions:**

We propose the ancestral *SLEEPER* gene was formed after a process of retro-transposition during the evolution of the first angiosperms. It may have acquired an important function early on, as mutation of two *SLEEPER* genes in rice, like the *daysleeper* mutant in *A. thaliana* gave a developmental phenotype indicative of their importance for normal plant development.

## Background

The role of transposons in evolution has long been greatly underestimated. Viewed as genomic parasites, transposons were classified as part of the so-called “junk-DNA” and largely ignored, even though transposons and transposon-remnants make up significant fractions of eukaryotic genomes [1]. Forty four percent of the human genome and more than 85% of the maize genome consists of transposons and their relics [2,3]. New views have led to the insight that transposons have shaped the genomic landscape in almost every conceivable way: shuffling, addition and deletion of not only new coding and regulatory sequences, but of large stretches of chromosomes as well [4,5].

Although a more detailed classification system is now being used, two major classes of transposable elements (TE’s) exist: retrotransposons, which transpose by using a RNA intermediate, and DNA transposons, which transpose by cutting their genomic sequence and inserting it elsewhere in the genome. These TE’s are referred to as “copy-paste” elements and “cut-paste” elements, respectively [1]. Retrotransposons encode several proteins that are highly similar to those encoded by retroviruses. One of these proteins is a reverse-transcriptase that is able to reverse-transcribe the full-length transposon mRNA into DNA, after which the new copy is integrated in the genome [1]. DNA transposons encode proteins, called transposases, which are able to cut their own coding sequence from the genomic DNA, by recognizing flanking repeats, and inserting it elsewhere in the genome. High transposon activity would be deleterious for the host and therefore defense mechanisms have evolved to counteract transposase activity. Still, transposons are numerous in almost every eukaryotic genome and thus have successfully managed to sustain themselves [6].

Transposons have contributed greatly, not only to shaping the genomic landscape, but also to the coding material of endogenous genes, for instance by giving rise to chimeric proteins (reviewed in [5]). Many conserved protein domains have now been shown to originate from transposable elements (e.g. BED zinc finger domains) [7]. In the process called “domestication” a transposase loses its original function and acquires new functionality, creating a novel gene. Various genes in different species have been found to be domesticated transposases (reviewed in [8]). A recurrent theme in domestication seems to be the conversion of transposases encoded by DNA transposons into important host proteins such as chromatin-related proteins and transcription factors. Among these factors are CENP-B, a centromere protein in vertebrates and fungi, the *FAR1-FHY3* family, involved in far-red light signaling in plants and BEAF-32, a boundary element associated factor in *Drosophila melanogaster*[5,7,9,10]. These elements are derived from, pogo, MuDR and hAT super-families of “cut-paste” elements respectively. This evolutionary trend can be explained by the fact that the transposases of these elements all contain DNA binding domains and protein-protein interaction domains, since they work in conjunction with host factors to enable the transposition process [11]. It seems likely that host partners of these transposases include chromatin remodelers, DNA repair genes and/or endonucleases, since one can envisage players in these fields to be required for facilitation of the “cut-paste” process. Remarkably, very little is known about these potential factors and the steps of the transposition process.

DAYSLEEPER was first described in 2005 by Bundock and Hooykaas [12]. The *DAYSLEEPER* gene in *Arabidopsis thaliana* is an example of molecular domestication of a DNA transposon. *DAYSLEEPER* shares extensive homology with members of a large subfamily of transposable elements, the hAT transposons, which are widely spread throughout the tree of life and are found in all eukaryotic branches, except in *Trichomonas*, diatoms, and ciliates [6]. Unlike these elements, *DAYSLEEPER* is not able to transpose, since it lacks the hallmark repeats essential for this process. Also, a number of amino acids shown to be essential for the transposition of the Ac-element, the first described hAT transposon family member of maize, are not conserved in DAYSLEEPER [12]. DAYSLEEPER was found to be essential to *Arabidopsis thaliana*, as displayed by a severe developmental phenotype in *daysleeper* mutants. The gene most likely codes for a DNA-binding protein, since it was identified through binding to the promoter of the DNA repair gene *Ku70* in a yeast one-hybrid assay [12]. DAYSLEEPER consists of 696 amino acids, possesses a DNA binding BED-type zinc finger domain and a hAT dimerization domain [12,13].

Here we present data on the presence of putative *DAYSLEEPER* orthologs in angiosperms, including the basal angiosperms. We show that *SLEEPER* genes are present in many species, often in multiple copies. Furthermore, we postulate a theory on the domestication process of the ancestral *SLEEPER* gene.

## Results

### *DAYSLEEPER* orthologs in the genome of *oryza sativa* and *vitis vinifera*

**Figure 1:**
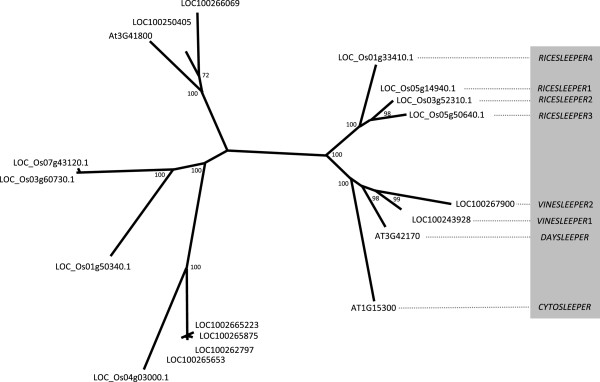
**Phylogeny of DAYSLEEPER homologs from Arabidopsis, rice and grapevine.** Rice sequences were obtained [15] and supplemented with the most homologous sequences from both the *Oryza sativa, Vitis vinifera* and the *Arabidopsis thaliana* genomes, found in genomic databases using TBLASTN queries. Gene identicators starting with “LOC_Os” “LOC10” or “At” indicates genes from rice, grapevine and Arabidopsis, respectively. Phylogeny was created using RAxML, with bootstrap values [21].

**Figure 2:**
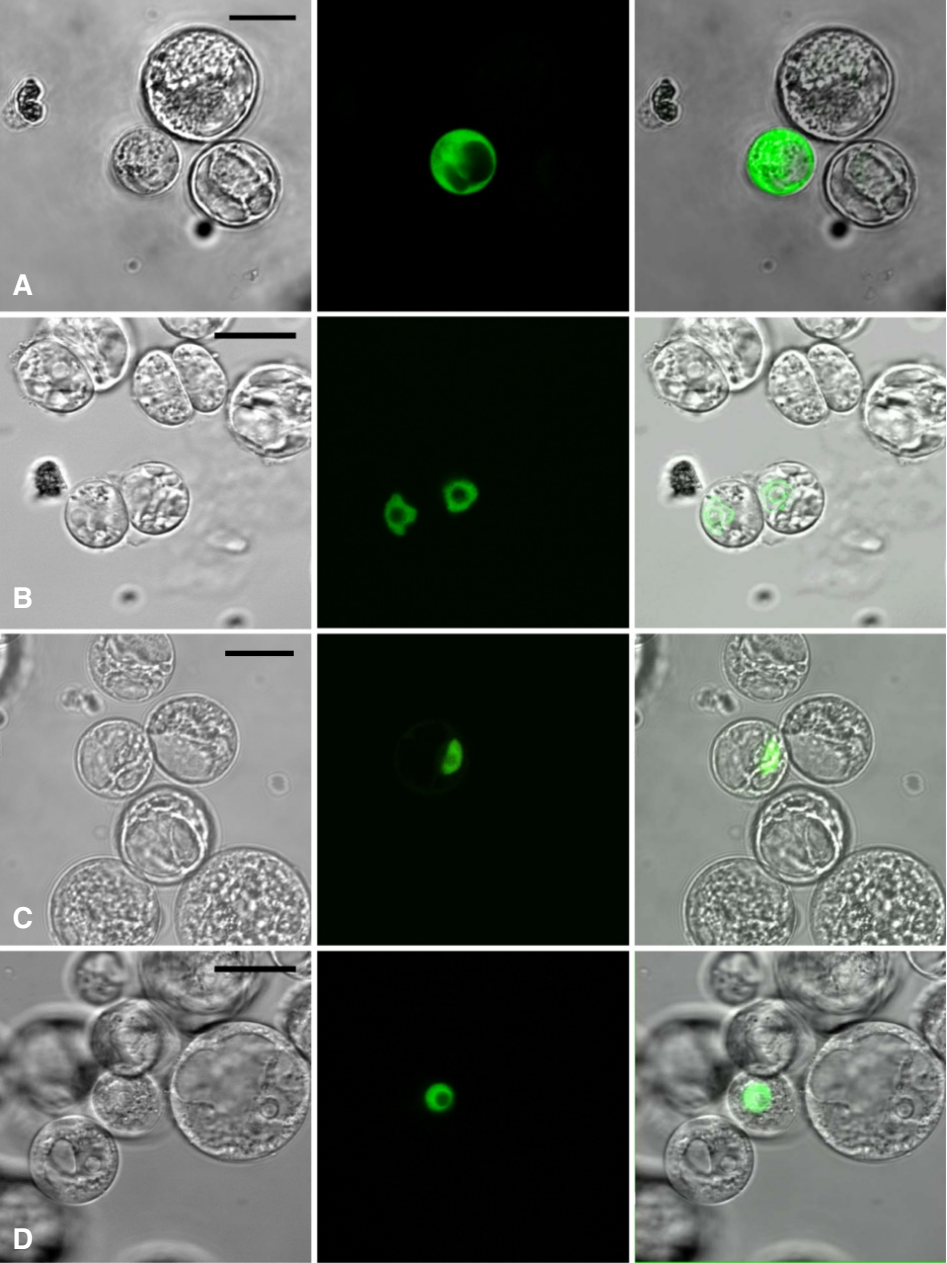
**Subcellular localization of SLEEPER proteins from different species in *Arabidopsis thaliana *cell-suspension protoplasts.A**. CYTOSLEEPER:YFP. **B**. VINESLEEPER2:YFP. **C**. RICESLEEPER3:YFP. **D**. DAYSLEEPER:YFP. Images in the left column are bright field images, the middle column depicts the fluorescent image and the right column merged images of the other two columns. The scale bar represents 20 μm.

Two genes that are possibly orthologous to *DAYSLEEPER*, were identified by Benjak et al. (2008) [14] in a genome-wide analysis of hAT-transposons in the grapevine genome and named *VINESLEEPER*1 and 2. In a study on the transcriptional activity of transposons in rice, several sequences were designated as “*DAYSLEEPER*-like” [15]. We used the DAYSLEEPER sequence as a query to find the most related sequences in the genomes of Arabidopsis, grapevine and rice and produced a maximum-likelihood phylogenetic tree with bootstrap values, depicted in Figure 1, to reveal the relationship between the highest scoring BLAST hits in the Arabidopsis, grapevine and rice genomes. This resulted in a clustering of putative *SLEEPER*s among the hAT-like transposase genes. The four putative orthologs we found in the rice genome only partly overlap with the *DAYSLEEPER*-like sequences reported by Jiao et al. (2007) [15]. Because of their high identity *RICESLEEPER* 1 and 2 are probably the result of a recent duplication event (Figure 1). One gene in *Arabidopsis*, At1g15300, was found to be related to *DAYSLEEPER*. A homozygous T-DNA insertion mutant (SALK_020839C) for this gene showed normal development. This gene is expressed, but may have become non-functional by a lack of the N-terminal R/K rich nuclear localization signal which is characteristically present in *DAYSLEEPER* and all *RICE-* and *VINESLEEPER*s or has acquired novel functionality. In order to determine their cellular localization, YFP-fusions were constructed for these *SLEEPER* genes and introduced in Arabidopsis protoplasts. This revealed a nuclear localization for all *SLEEPER*s from rice, grapevine and Arabidopsis, but not for the product of the At1g15300 gene, which is present in the cytosol and which we therefore named *CYTOSLEEPER* (Figure 2).

### SLEEPER structure and conserved domains

**Figure 3:**
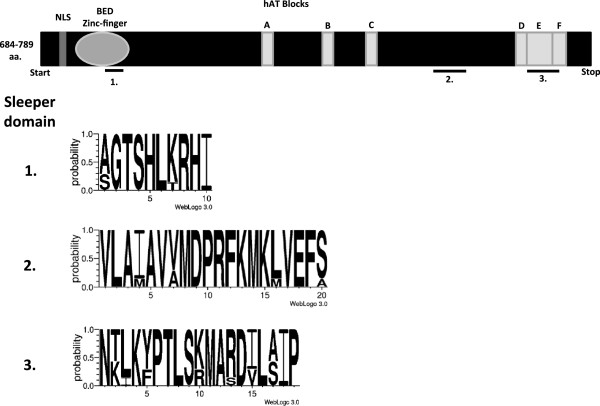
**Schematic overview of the structure of hAT transposase genes.** hAT transposase genes possess an N-terminal NLS, followed by a BED-type zinc finger domain and conserved hAT-blocks **A** until **F**. The last three hAT blocks (**DF**) make up the hAT dimerization domain. Three highly conserved motifs (1–3) were identified by aligning all SLEEPERs from *Arabidopsis thaliana, Vitis vinifera* and *Oryza sativa.* SLEEPERmotifs are depicted using Weblogo 3.0 [40].

We found that genes coding for SLEEPERs are conserved between different species. SLEEPERs contain hAT motifs that are widely conserved in hAT transposases. Six hAT motifs are generally found in hAT transposases from various species, which are named motif A to F [16]. SLEEPERs contain a K/R rich nuclear localization domain (NLS) adjacent to a BED-type zinc finger at their N-terminal region and have a hAT transposase-like dimerization domain at the C-terminus (Figure 3) [12,16]. Like hAT transposases, SLEEPERs are generally present in the nucleus (Figure 2B,C,D). In DAYSLEEPER, the C-terminal dimerization domain is functional as well (M. Knip, unpublished results), allowing DAYSLEEPER to homodimerize. Like DAYSLEEPER, RICE- and VINESLEEPERs lack the amino acids necessary for transposition and the genes are not flanked by hAT repeat sequences (data not shown).

The SLEEPER*s* form a separate group when compared to other hAT-transposases (Figure 1). SLEEPERs contain three strongly conserved motifs, that we designated SLEEPERmotifs1 to 3 (Figure 3). SLEEPERmotif1 encompasses part of the BED-zinc finger, raising the possibility that SLEEPERs might bind conserved sites in different species. SLEEPERmotif2 does not overlap with any of the conserved hAT blocks and is, in contrast to SLEEPERmotif1 and 3, not exclusive to SLEEPERs, since BLAST searches using this motif also yield hAT transposases in various species that lack SLEEPERmotif1 and 3. SLEEPERmotif3 overlaps largely with hAT block E. This hAT block is part of the hAT dimerization domain, in conjunction with hAT block D and F, suggesting that other SLEEPERs can dimerize like DAYSLEEPER and other hAT transposases [13]. Localization of SLEEPERs is nuclear in Arabidopsis protoplasts, but CYTOSLEEPER, which lacks the K/R rich conserved array adjacent to the BED-zinc finger, is present in the cytosol, suggesting that this domain is indeed necessary for nuclear localization of SLEEPERs (Figure 2). The divergent sequence of CYTOSLEEPER, represented by the long branch-length in Figure 3, and the lack of an apparent phenotype in mutant plants indicate that this gene might be pseudogenized or has obtained a different function as DAYSLEEPER. *SLEEPER* genes from Arabidopsis, grapevine and rice, do not have introns in their coding sequences. Most other *SLEEPER* genes contain one intron between the 5’ UTR and their start-codons. DAYSLEEPER shares ~50% identity (61-69% similarity) with the VINESLEEPERs and between 36 and 43% identity with the RICESLEEPERs (51-58% similarity) at the amino acid level (Table 1). RICE- and VINESLEEPERs vary in length between 684 and 753 amino acids (Figure 3, Table 1). The increased length of RICESLEEPER4 is mainly caused by the acquisition of extra coding sequence at its N-terminus. The relatively large size of CYTOSLEEPER is predominantly due to an additional stretch of amino acids between the second and third of three conserved motifs, which is not found in other SLEEPERs.

**Table 1:** Homology of the VINESLEEPERs and RICESLEEPERs to DAYSLEEPER

	**Compared to DAYSLEEPER(696 AA’s)**		**Coding sequence length(AA’s)**
	**Identity positions (%)**	**Consensus positions (%)**	
**CYTOSLEEPER**	30.1	42.1	799
**VINESLEEPER1**	48.4	60.7	689
**VINESLEEPER2**	55.9	68.5	675
**RICESLEEPER1**	43.0	58.1	722
**RICESLEEPER2**	43.3	58.0	722
**RICESLEEPER3**	35.7	51.4	684
**RICESLEEPER4**	37.4	53.8	752

Similarity and identity values, as well as the length of the SLEEPER, are depicted. The numbers were obtained using AlignX in the Invitrogen Vector NTI suite (Invitrogen®).

### *SLEEPER*s are only present in higher plants

An important question is where and when the SLEEPERs have emerged in evolution. To answer this question SLEEPERmotif1 and 3 consensus sequences and DAYSLEEPER were used in TBLASTN searches in genomic and EST databases from several organisms. Queries with the SLEEPERmotifs yielded exclusively high-scoring hits for *SLEEPER*-like sequences in the monocotyledonous (Poaceae) and dicotyledonous species searched. In databases of species beyond the plant realm, namely *Saccharamyces cerevisiae* and *Drosophila melanogaster*, no similar sequences were found (standard settings: Max target sequences = 100, expect threshold = 10, word size = 3, NCBI BLAST [17]). Also, the EST library for gymnosperm species *Ginkgo biloba* (data not shown) and a mixed *Pinus*-species library (TIGR plant transcript assemblies [18]) did not yield any significant hits (Additional file 1: Table S1), neither did queries in databases of the lycophyte *Selaginella moellendorffii* (Additional file 1: Table S1) and the moss *Physcomitrella patens* (Phytozome [19]) (data not shown). However, lower angiosperm EST databases (Ancestral Angiosperm Genome Project; http://ancangio.uga.edu/content/est-assemblies) yielded hits in several species of different orders, namely *Persea americana* (order: Laurales), *Liriodendron tulipifera* (order: Magnoliales), *Nuphar advena* (order: Nymphaeales) and *Amborella trichopoda* (order: Amborellales) (Table 2). These data indicate that *SLEEPER* genes belong to an angiosperm specific gene family and that formation of the first *SLEEPER* gene coincided with the evolution of angiosperms.

**Table 2:** **Evidence of *SLEEPER *gene expression in lower Angiosperms**

**Species**	**Sleeperdomain 1**.	**Sleeperdomain 3**.	**Full length DAYSLEEPER**
***Persea americana***	b4_ep_c61270, b4_c39392	b4_c14697, b4_c9266, b4_ep_c32228	b4_c2641, b4_c7656
***Nuphar advena***	b3_c39269	b3_c17103, b3_c9604	b3_c707, b3_c1078
***Liriodendron tulipifera***	b3_c3339, b3_c108364	b3_c2953, b3_c39743	b3_c2953, b3_c21053
***Amborella trichopoda***	b4_c220959, b4_c97395	b4_c12734	EST hits too short

TBLASTN searches were performed on the EST databases of the AAGP (Ancestral Angiosperm Genome Project; http://ancangio.uga.edu/). Only unique ESTs are shown. The cut-off score for ESTs found with the full-length *DAYSLEEPER* sequence TBLASTN query is 400. Queries were performed with *SLEEPER*motif1 and 3 and the full-length amino acid sequence of DAYSLEEPER.

### *SLEEPER*s are frequently copied in several species

**Figure 4:**
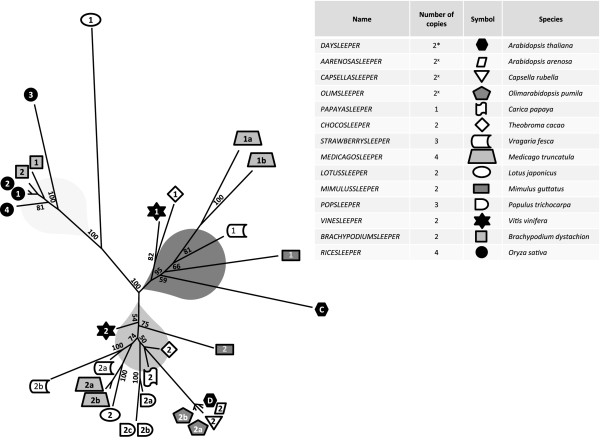
**Phylogenetic tree depicting *SLEEPER *genes from various species.** Dark grey = *CYTOSLEEPER* cluster. Grey = *DAYSLEEPER* cluster. Light grey = Poaceae cluster. Sequences that were used for complementation studies have a black logo. * Contains 1 *DAYSLEEPER* gene (D), and *CYTOSLEEPER* (C). ^X^ These species contain one *DAYSLEEPER* ortholog (shown) and a *CYTOSLEEPER* ortholog (not depicted). The number inside the symbol is the number assigned to each gene. The tree is created from protein sequences aligned with ClustalW [33], processed by the RaxML algorythm, with bootstrap values enabled [21]. Clusters have been given a color.

TBLASTN searches using the amino acid sequence of DAYSLEEPER in genomic databases of several sequenced angiosperm species (Figure 4), revealed that *SLEEPER* genes are present in all these queried genomes and often in multiple copies (Plant Genome Database [20]). Figure 4 depicts a maximum likelihood-tree with bootstrap values, generated with the RAxML algorithm [21]. Many genomes appear to have several *DAYSLEEPER* homologs. *SLEEPER* genes possess the three SLEEPERmotifs and were distinguished from hAT transposase sequences by a BLAST score of over 400, whereas hAT-like sequences typically did not score higher than 200.

It is clear to see a clustering of *SLEEPER* genes from Poaceae, separated from those of dicotyledonous plants, which form two groups, grouping with either *CYTOSLEEPER* or *DAYSLEEPER* (Figure 4). *LOTUSSLEEPER*1 is exceptional in that it has diverged rather far from the other *SLEEPERs* in dicotyledonous plants. Since *VINESLEEPER*1 and 2 were described by Benjak *et al*. [14] and these proteins cluster in separate groups, we decided to use a similar naming scheme for all *SLEEPER*s. We found synteny between the genomic regions in which the *VINESLEEPER2* and *DAYSLEEPER* genes reside, suggesting they are homologs (Additional file 2: Figure S1). Although high similarity exists between RICESLEEPERs, we chose to designate the RICESLEEPERs with individual numbers, namely 1 to 4. The coding sequence of *RICESLEEPER*1 and 2 are almost identical (97% sequence identity), as are *RICESLEEPER*3 and 4, *OLIMSLEEPER*2a and 2b and *POPSLEEPER*2b and 2c. These may therefore be relatively recent duplications, which had been shown previously for the genes in *Olimarabidopsis pumila* by Hall et al. [22]. In dicotyledonous plants, all recent duplications seem to have occurred in the *DAYSLEEPER*-branch of the phylogeny shown in Figure 4. When looking closer at the rice genome, there is no evidence for a segmental duplication of the genomic location of the *RICESLEEPER*1 and 2 genes, since there is no apparent sequence homology or synteny of the region surrounding these genes. The close relatives of *Arabidopsis thaliana,* namely *Olimarabidopsis pumila*, *Arabidopsis arenosa* and *Capsella rubella*, have homologs of the *CYTOSLEEPER* gene, but these genes are not depicted in the phylogeny, since the complete genome sequence of these species was not available at the time of the analysis (Figure 4).

Unlike *CYTOSLEEPER,* genes clustering with *VINESLEEPER*1 do code for a K/R-rich putative nuclear localization domain. Most dicotyledonous species analyzed also have a homolog in both the *CYTOSLEEPER,* as well as the *DAYSLEEPER* cluster (Figure 4). Exceptions are poplar, which has three *POPSLEEPERS* clustering with *DAYSLEEPER, Lotus japonicus*, which has *LOTUSSLEEPER*2 clustering with *DAYSLEEPER* and *LOTUSSLEEPER*1, which has diverged from other *SLEEPER*s and *Carica papaya*, which apparently has only one *SLEEPER*. This might suggest that *SLEEPER*s clustering with *DAYSLEEPER* are functionally more conserved than *CYTOSLEEPER*-clustering *SLEEPER*s. It has to be noted that two auxiliary *SLEEPER-*like genes were identified in *Carica papaya*. These genes showed BLAST (TBLASTN) values of just below 400 in relation to *DAYSLEEPER* and did not possess a conserved SLEEPERmotif1. These genes were therefore not included in Figure 4. If they were included in the alignment, these sequences cluster with *LOTUSSLEEPER*1, albeit with very long branch-length (data not shown).

### *RICE- and VINESLEEPER* cause a dominant phenotype when expressed in Arabidopsis

**Figure 5:**
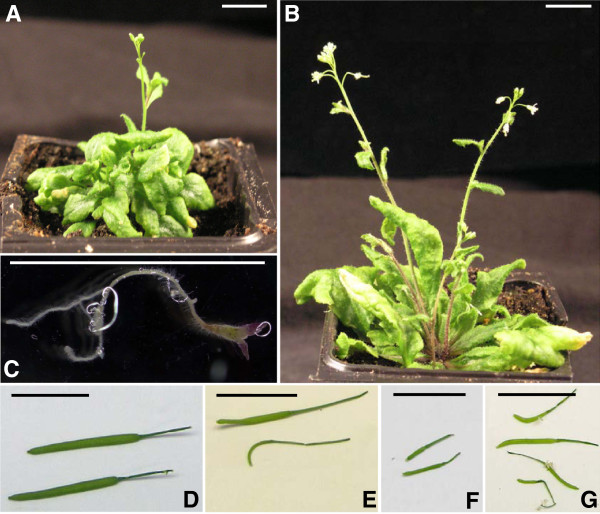
**Phenotype of Arabidopsis plants expressing VINE- or RICESLEEPERs. A**. *DAYSLEEPER*^+/−^ plant expressing *pDAYSLEEPER::RICESLEEPER4*. **B**. *DAYSLEEPER*^+/−^ plant expressing *pDAYSLEEPER::RICESLEEPER3*. **C**. *daysleeper* mutant harboring *pDAYSLEEPER::VINESLEEPER1:HA*. **D**. Siliques from Col-O plants **E**. Siliques from *DAYSLEEPER*^+/−^ plant expressing *pDAYSLEEPER::VINESLEEPER2*. **F**. Siliques from *DAYSLEEPER*^+/−^ plant expressing *pDAYSLEEPER::RICESLEEPER3*. **G**. Siliques from *DAYSLEEPER*^+/−^ plant expressing *pDAYSLEEPER::RICESLEEPER4*. Plants depicted in **A** and **B** are 8 weeks old. The scale bars represent 1 cm.

To assess functionality of the *SLEEPER* genes found in other species, we attempted to complement the *daysleeper* phenotype with coding sequences from rice and grapevine under control of the 3.6 kb upstream region of *DAYSLEEPER*, including the 5’UTR. We found that the *daysleeper* phenotype cannot be complemented by these constructs, although we were able to restore the wild-type phenotype with *GFP:DAYSLEEPER* constructs. We found seedlings with the *daysleeper* phenotype despite the presence of either one of the *RICESLEEPERS* (Figure 5C) or *VINESLEEPERS* (not shown).

Interestingly, the complementation constructs did invoke a dominant phenotype in Arabidopsis plants with the *DAYSLEEPER*-gene still present. Such plants made an excess of rosette leaves, often curled, and were delayed in formation of inflorescences and in flowering (Figure 5A,B). Furthermore, these plants formed small siliques, suggesting issues with seed development (Figure 5D-G). Interestingly, we did not observe differences between plants containing the various constructs. However, we did observe differences in phenotype severity among plants that were direct descendants of a primary transformant (data not shown). This suggests that the observed phenotype is associated to *SLEEPER* abundance, influenced by DAYSLEEPER hetero- or homozygosity or the number of T-DNA inserts. *DAYSLEEPER* overexpression under control of the strong 35S promoter results in a similar phenotype as described above [12], also we observed similar phenotypic traits in some plants when trying to complement *daysleeper* mutant plants with a *GFP:DAYSLEEPER* construct (data not shown). Complementation of *daysleeper* was not found with the coding sequence of At1g15300 (*CYTOSLEEPER*) under control of the *DAYSLEEPER* promoter region. Multiple plants of four individual T -DNA insertion lines were extensively analyzed, but none of these revealed a rescue of the *daysleeper* phenotype, or resulted in *DAYSLEEPER* overexpression-like phenotypes.

### *RICESLEEPER*1 and *RICESLEEPER2*

**Figure 6:**
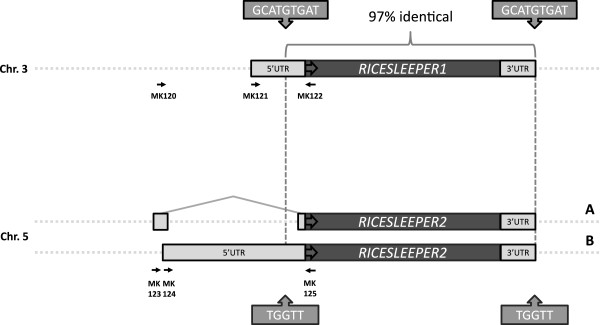
**Comparison of the *RICESLEEPER*1 and 2 loci.** Coding regions and part of the 5’and 3’UTR’s are ~97% identical. *RICESLEEPER*1 and 2 each have obtained a new 5’UTR sequence, which is not homologous to that of the other locus. **A** and **B** display two different transcription models found by PCR for *RICESLEEPER*2. Transcription model B has most likely acquired sequence material from a retrotransposon insertion in an intron on the opposite strand. Short duplications were found flanking the zones of homology in both genes, which are shown in arrowed boxes. The small arrows represent PCR primers, which were designed on available rice gene expression data, and were used to obtain *RICESLEEPER*1 and 2 transcripts from a cDNA library. Primer descriptions can be found in Additional file 3: Table S2.

*RICESLEEPER* 1 and 2 have nearly identical coding sequences and probably both have arisen from relatively recent duplication events. A comparison between the *RICESLEEPER*1 and 2 loci can be seen in Figure 6. *RICESLEEPER*2 is predicted to have an intron in its 5’ UTR, whereas *RICESLEEPER*1 is predicted to be intronless. To verify these predictions, we designed primers based on available mRNA and EST sequences and tried to amplify the 5’ UTR from rice cDNA (PlantGDB [20] and GenBank) (Additional file 1: Table S1). The PCR-fragments we obtained were isolated and sequenced. We found two different transcripts for *RICESLEEPER*2, which we named “A” and “B” (Figure 6). Gene model A corresponds with the predicted transcript (Rice Genome Browser [23]), whereas the transcript depicted in gene model B contains an unspliced UTR that stretches to ~1500bp upstream of the start codon (Figure 6). For the *RICESLEEPER*1 gene, no UTR’s other than the predicted intronless 574 bases directly adjacent to the start codon could be amplified.

To study whether *RICESLEEPER* mutation would result in similar developmental defects as seen in the *A. thaliana daysleeper* mutant, two rice T-DNA insertion lines were obtained (Postech, Functional Genomics Laboratory) [24,25]. *RICESLEEPER*1 is disrupted by a T-DNA insertion in the coding sequence at approximately 1700 bp from the start codon (line: PFG_1D-01516). The T-DNA insertion in the *RICESLEEPER* 2 locus is located in the 3’UTR of the gene (line: PFG_1B-21919). Presence of the T-DNA was verified by PCR (data not shown, Additional file 3: Table S2).

**Figure 7:**
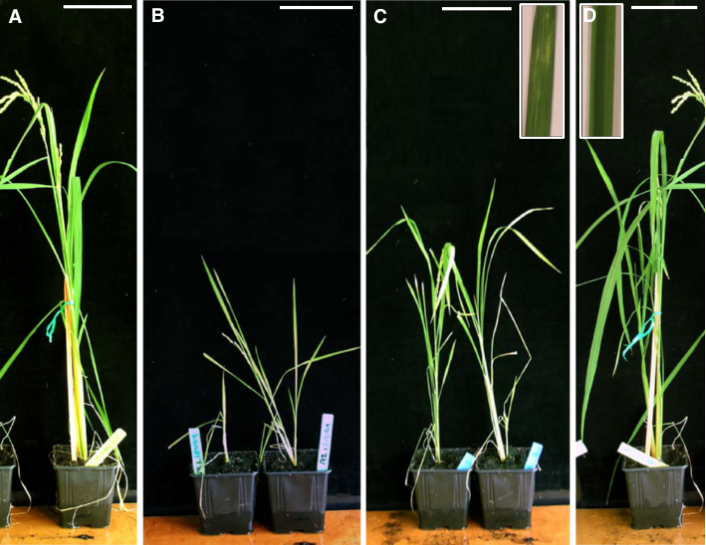
***RICESLEEPER*1 and *RICESLEEPER*2 T-DNA insertion mutants.A**, **D**. Wild-type plants of cultivars Dongjin and Daesan, respectively. **B**. *RICESLEEPER*2 insertion mutant plants (PFG_1B-21919, Dongjin cultivar). **C**: *RICESLEEPER1* insertion mutant plants (PFG_1D-01516, Daesan cultivar).The inserts in C, D show leaf sections of respectively a *RICESLEEPER1* insertion mutant plant and a wild-type cv. Daesan plant. Plants were photographed 80 days after germination. Scale bars represent 10cm.

Hygromycin resistant heterozygous seeds were obtained and grown and progeny of these plants analyzed. For both insertion lines only wild-type and heterozygous plants were identified, indicating that plants containing an insert in both copies of either *RICESLEEPER1* or *RICESLEEPER2* might be lethal at a very early stage. Hygromycin-resistant progeny of the *RICESLEEPER*2 insertion mutants reached about half the height of wild-type plants (Figure 7A,B). *RICESLEEPER*1 insertion mutants also remained somewhat smaller than wild-type plants (approximately two thirds of wild-type height), but not as small as *RICESLEEPER*2 mutants. *RICESLEEPER2* mutants produced a normal amount of seeds, but *RICESLEEPER*1 mutant plants produced mostly empty panicles, yielding only very few seeds (Figure 7C,D), indicating a lethal embryo defect. Organs of both insertion mutants developed normally. However, yellow discolorations were observed in *RICESLEEPER1* mutant plant leaves (Figure 7C insert), which are not present in wild-type plants (Figure 7D insert), or *RICESLEEPER2* mutant plants (not shown).

## Discussion

### *DAYSLEEPER* conservation

All SLEEPERs have highly conserved features in the form of their N-terminally located BED-zinc finger DNA binding domain, flanked by a nuclear localization domain and the C-terminal dimerization domain. These partly overlap with SLEEPERmotif1 and 3 respectively, whereas SLEEPERmotif2 is localized adjacent to the dimerization domain, but has no overlap or homology to any known functional domain or motif. The *CYTOSLEEPER* gene seems to be a divergent homolog of *DAYSLEEPER*. CYTOSLEEPER possesses the SLEEPERmotifs, but has lost its nuclear localization signal, which is highly conserved in other SLEEPERs. This sequence divergence and the lack of the nuclear localization motif might indicate pseudogenization. CYTOSLEEPER has relatively well conserved SLEEPERmotifs and phylogenetically clusters with the SLEEPERs (Figure 1), but its amino acid sequence is only 30.1% identical to DAYSLEEPER (Table 1). A homozygous insertion mutant (SALK_020839C) displays no phenotype and its coding sequence cannot complement the *daysleeper* phenotype. However, it seems likely that *CYTOSLEEPER* has acquired novel functionality, since it seems that a selective pressure exists to maintain *CYTOSLEEPER*. We calculated the ratio of the number of non-synonymous substitutions per non-synonymous site (Ka) to the number of synonymous substitutions per synonymous site (Ks), to determine if selection pressure exists to maintain *CYTOSLEEPER.* Ka/Ks ratio (0,29) is similar to that of *DAYSLEEPER* (0,28), when comparing these genes in *Arabidopsis thaliana* and *Capsella rubella* (Additional file 4: Figure S2).

The highly conserved DNA-binding domain, which spans the location of the second α-helix of the BED-zinc finger [7], might hint to a conserved recognition sequence for all SLEEPERs. SLEEPERmotif 3 is located in the dimerization domain of the SLEEPER coding sequence. The dimerization domain is essential for DAYSLEEPER function, since a C-terminal truncation lacking this domain is not able to rescue the *daysleeper* phenotype (M. Knip; unpublished results). The high conservation of the dimerization domain in *SLEEPER* genes also offers the theoretical possibility of heterodimerization between SLEEPERs, for instance in the case of DAYSLEEPER and CYTOSLEEPER. Heterodimerization can theoretically take place, since expression patterns of these genes overlap in several tissues (Arabidopsis eFP-browser [26], data not shown). The possibility of heterodimerization is even likely in the case of RICESLEEPER1 and 2, since their coding sequences are almost identical and their expression patterns partly overlap [23] . We have found that nuclear heterodimerization is possible *in vivo* for DAYSLEEPER and RICESLEEPER4 (Figure 2) in a Bi-molecular fluorescence complementation (BiFC) assay in Arabidopsis protoplasts, using DAYSLEEPER:YC and YN:RICESLEEPER4 fusion proteins (data not shown). The ability to heterodimerize may offer an interesting layer of complexity to the function of SLEEPER proteins in several species.

### *SLEEPER* complementation

Although complementation of *DAYSLEEPER* is not found with constructs containing other *SLEEPER*s, these constructs cause a dominant phenotype in Arabidopsis (Figure 5). The transformed plants display developmental issues: delayed formation of the inflorescence and irregular and increased formation of leaves, fasciation and dwarfism have been observed in all lines. This phenotype resembles the overexpression phenotype of plants bearing a *35S:DAYSLEEPER* construct [12] and it is probable that this effect is caused by increased expression of *SLEEPER* genes in these plants. This is further substantiated by the fact that mild overexpression phenotypes were also observed in some *daysleeper* mutant plants complemented with a *GFP:DAYSLEEPER* construct (data not shown). The fact that *SLEEPER*s cause this phenotype suggests that they are at least partially functionally similar to *DAYSLEEPER*. Interestingly, the clustering of CYTOSLEEPER with other SLEEPERs, such as VINESLEEPER1, suggests that other species possess functional SLEEPERs that are derived from the same duplication as the *CYTOSLEEPER* gene. In poplar, none of the *SLEEPER* genes found cluster with *CYTOSLEEPER*, suggesting that a *SLEEPER* derived from the duplication event mentioned above, was lost in this species.

### *RICESLEEPER *1 and 2

*RICESLEEPER*1 and 2 are highly similar and have arisen from a duplication event (Figure 6). We suggest that these *RICESLEEPER* genes are relatively recently duplicated retrogenes. In the rice genome many retrocopies and retrogenes can be found, which could be explained by the overall high activity of LTR retrotransposons in this species [27]. Retrocopied genes are devoid of introns, since they are derived from mRNA sequences and are flanked by short non-transposon-derived duplications. Both *RICESLEEPER*1 and 2 meet these criteria (Figure 6). Recent retrocopies often possess a relic poly-A tail, derived from the mRNA they originated from [28]. Both *RICESLEEPER* genes lack a clear poly A-tail. However, this feature is lost in many retrocopied genes, notably those derived from older retrocopy events [29,30]. Like other SLEEPER-proteins, RICESLEEPER1 and 2 lack the amino acids necessary for transposition and are not flanked by the characteristic hAT features (data not shown) [16]. Transcription of the 5’ UTR of both genes starts before the site where the genes become highly similar. It is thought that retrocopies can acquire new (non-)coding material from their site of insertion in the genome, or by secondary sequence insertions upstream, in a process called exonization (Figure 6). Exonization seems to have taken place at the *RICESLEEPER*2 locus. The found 5’ UTR of *RICESLEEPER*2 (depicted in model A. of Figure 6) largely overlaps with the first exon of a Ty3/Gypsy-like retrotransposon gene (LOC_Os05g14950.1) which is predicted to be situated on the opposite strand. The parental template gene of *RICESLEEPER*1 and 2 was not identified in the rice genome. This leaves the possibility that either *RICESLEEPER*1 or 2 has been retrocopied to give rise to *RICESLEEPER*2 or 1, respectively. This would imply that both genes have acquired new 5’ UTR sequences after the retrocopy event, or that a partial mRNA served as a retrocopy template. A model of how we think the ancestral *SLEEPER*s could have become domesticated will be discussed below. This model also includes exonization of coding material from a TE insertion, which may have happened in the *RICESLEEPER*2 locus. *RICESLEEPER*1 and 2 are differentially expressed, and mutants of these genes give rise to different phenotypes (Figure 7). We suspect the divergent expression patterns and/or the difference in the non-coding parts of their transcripts attribute to the differences which these genes play in the rice plant.

### *SLEEPER* domestication

*SLEEPER* genes are domesticated transposase genes, but the mechanism of domestication remains undetermined. We propose that the ancestral domesticated *SLEEPER* gene was the result of a retroposition event. We suggest that the ancestral *SLEEPER* gene is the product of a hAT transposase transcript being reverse-transcribed and integrated somewhere in the genome. The site of integration provided the retrocopy with regulatory elements and UTR material, either by a secondary insertion of a TE upstream or by sequences already present, turning the copy into a functional retrogene (Additional file 5: Figure S3). We base our model mainly on the fact that all *SLEEPER* genes studied are without introns in their coding sequence, as opposed to hAT transposase-genes, which typically contain introns (Table 3). This is especially noteworthy in the light that also the most *SLEEPER*-related hAT transposase-annotated genes that are expressed in both Arabidopsis and *Oryza sativa,* contain one or several introns in their coding sequence, based on EST evidence (Table 3). The mRNA-derived poly-A tail, a feature of retrocopies, is lost over time by sequence erosion or a deletion event in the *SLEEPER* family, which has been reported to happen in many retrocopies [27,29]. Analysis of the SKP1 gene family in angiosperms, for instance, revealed several retroposition events, but only one retrogene that possessed a poly-A tail was identified [30]. Obvious poly-A tails can also not be found in any of the *SLEEPER* loci in the grapevine and rice genomes. It is not surprising that poly-A tails from these domesticating retroposition events are not present anymore, since the origin of *SLEEPER*s is apparently timed when gymnosperms and angiosperms separated (~228Myr ago) and most likely no selection pressure was exerted to maintain these short sequences [31]. The short duplications flanking recent retrocopy events, like the sequences found at the *RICESLEEPER*1 and 2 loci, have eroded in other *SLEEPER*s (Figure 6). Genome information of lower angiosperms and gymnosperms could facilitate a more in depth sequence analysis, but these sequence data were not available at the time of this study. Ty1-copia elements have been found to be active in several gymnosperm species, potentially facilitating the creation of retrogenes [32].

**Table 3:** **Expressed hAT**-**like genes in *Arabidopsis thaliana *and *Oryza sativa***

***Arabidopsis thaliana***	**Introns in CDS**	***Oryza sativa***	**Introns in CDS**
AT1G80020.1	1*	LOC_Os04g53660.1	1
AT3G14800.1	2	LOC_Os03g60730.1	1
AT4G13120.1	2	LOC_Os01g50340.1	1
		LOC_Os07g43120.1	3

hAT-like genes, closely related to *SLEEPER*s contain at least one intron in their coding sequence. Most genes also possess 2 introns in their UTR’s, which were not included in this table. ”*” Indicates an EST showing the presence of an intron, but without a predictive gene model in the TAIR genome browser.

All the evidence indicated above, together with the fact that we have found signs of a recent retrocopy event in the form of *RICESLEEPER*1 and 2 suggests that a retrocopy event may be responsible for the domestication of *DAYSLEEPER*. Although alternative scenarios are conceivable, we think our model provides an elegant way for a transposase gene to shed its repeats and start a new, stable life elsewhere in the genome.

## Conclusions

We found that *SLEEPER*s have conserved features and are often duplicated. We show that SLEEPER genes are an angiosperm-specific gene family, and that early in dicotyledon evolution two copies of *SLEEPER* genes were present. The *SLEEPER* family is an intriguing example of how transposons can give rise to new genes. Analysis of the phylogeny of the *SLEEPER*s reveals the dynamic interplay between transposons. In recent years many ways of shaping the genome by TE’s have been described, and it seems without doubt that many more new genes derived from TE’s and evolutionary effects of TE’s will be uncovered in the coming years. The presence of *SLEEPER* genes in many species and the severe *daysleeper* phenotype in Arabidopsis are testimony to their importance in higher plants. We show that the *SLEEPER* gene-family is angiosperm specific and that *SLEEPER*s have become important genes in these plants, as was confirmed in rice, where T-DNA insertions in *SLEEPER* genes gave rise to aberrant phenotypes. Future studies may reveal the molecular mechanisms underlying the functional role of *DAYSLEEPER* and its orthologs in plant development.

## Methods

### Genome browsers and BLAST databases

Genome browsers for *Arabidopsis thaliana (*TAIR; http://www.arabidopsis.org*)*, *Oryza sativa* and *Vitis vinifera* (Genoscope; http://www.genoscope.cns.fr) were used for finding synteny in genomic regions and for visualizing (predicted) the various *SLEEPER* genes [23]. Genomic BLAST searches were performed at the NCBI website for the *Arabidopsis thaliana* and *Oryza sativa* genome [17]. The Genoscope BLAST Server was queried for *Vitis vinifera* (Genoscope; http://www.genoscope.cns.fr). Genetic information and BLAST searches for other species were performed at the PlantGDB website [20]. The standard BLAST settings were used at al websites. Word-size and the Expect-parameter were decreased to “3” and “10” respectively to be able to find shorter and/or more divergent sequences.

### Alignments and phylogenies

Alignments were created and edited using JalView 2.4 and processed using the integrated ClustalW function [33,34]. Phylogenies were created using the RAxML algorithm as offered by the RAxML-blackbox, using amino acid alignments [21]. Bootstrap values were calculated and the number of calculated trees was automatically determined by the RAxML algorithm. The generated phylogenies were graphically edited using FigTree v1.3.1 (Andrew Rombaut, University of Edinburgh) and Microsoft Office Powerpoint 2010 (Microsoft ®). The TIRfinder program was used to scan sequences for terminal inverted repeats flanked by host duplications. TIRfinder was run using the same settings as in Rubin et al. 2001 [16]. Relaxed settings were used to confirm the absence of the mentioned repeat sequences. Parameter “Tir_length” was set to minimal length of 7 and maximal length of 10. The direct repeat parameter (“Dir_length”) was set with a minimum of 7 and a maximum of 10 and allowing a distance of 15bp [16].

### Identification and isolation of *SLEEPER* genes from *vitis vinifera, oryza sativa* and *Arabidopsis thaliana*

Using TBLASTN searches expressed orthologous genes were found in the genome of *Arabidopsis thaliana*, *Oryza sativa* and *Vitis vinifera* (See “Genome Browsers and BLAST Databases”). None of the orthologs contained any introns in their coding sequences (CDS). The CDS of all genes were amplified from start (ATG) to stop codon, with genomic DNA as a template. Amplicons were cloned into pJET1.2 (Fermentas®) and sequenced.

### Cloning

Using PCR, with primers MK98 and MK99, the gateway cassette of pEARLEYGATE302 (ABRC; http://www.arabidopsis.org), containing the FLAG sequence and the T_NOS_ were isolated and cloned. This sequence, from now on referred to as “gateway® cassette”, was isolated, digested with HindIII and cloned into a pCAMBIA2300 vector (Cambia Australia®) (Additional file 1: Table S1) [35]. The resulting plasmid has a multiple cloning site (MCS) flanking the inserted gateway cassette. The MCS was used to insert a 3.8 kb stretch of upstream DNA sequence directly preceding the CDS of the DAY*SLEEPER* gene. Using PCR, with primers MK3.3 and MK9.3 the respective restriction sites SacI and KpnI were added to the promoter sequence (Additional file 1: Table S1) and were used to clone the fragment in the MCS of the vector, giving rise to the pCAMBIA2300 p*DAYSLEEPER* gateway FLAG T_NOS_ destination vector.

Subsequent cloning of the diverse *SLEEPER* sequences from different species was performed using the Invitrogen gateway technology, using pDONR207 (Invitrogen®) as the entry clone for the various coding sequences. Gateway compatible primers were designed to amplify the *DAY*-, *CYTO*-, *VINE*- and *RICESLEEPER*’s coding sequences without the stop codon (Additional file 1: Table S1). The obtained amplicon was recombined using the Gateway BP reaction into the pDONR207 vector (Invitrogen®) and the insert was sequenced. The obtained entry clones (pENTR) were recombined using the gateway LR clonase reaction into the pCAMBIA2300 p*DAYSLEEPER* Gateway FLAG T_NOS_ destination vector, described above (Invitrogen®). This lead to a translational fusion of the *SLEEPER* genes with a C-terminally fused FLAG-tag, under control of the *DAYSLEEPER* native promoter. Created plasmids can be found in Additional file 6: Table S3.

The *pDAYSLEEPER*::*DAYSLEEPER* sequence was isolated directly from genomic DNA with PCR using a forward primer MK43, binding 3.6kb upstream of the start codon and a reverse primer MK44 binding to the end of the *DAYSLEEPER* coding sequence (Additional file 1: Table S1). The resulting fragment was recombined into pDONR207 as described above and subsequently inserted into pEARLEYGATE302 using the Gateway LR clonase reaction (Invitrogen ®). The vectors used in the protoplast experiment (Figure 2) were created by using vector pART7 p35S gateway YFP:HA [36]. This vector was recombined using the pENTR vectors described above, using the LR clonase reaction, giving rise to a translational fusion of *SLEEPER*-genes and C-terminally fused YFP and HA-tag.

All PCR’s were performed using Phusion polymerase in HF buffer (Finnzymes®). Reaction conditions were as recommended, except for MgCl_2_, which was increased to 5,5 mM. The annealing temperature with Gateway®-compatible primers was set to 65°C (Invitrogen®). All obtained fragments were sequenced to check for PCR-induced errors. Primers are shown in Additional file 3: Table S2.

### Plant transformation

Binary expression vectors were electroporated into electrocompetent *Agrobacterium tumefaciens* strain AGL1 [37]. Floral dip transformation was performed with *Arabidopsis thaliana* Col-0 plants heterozygous for a T-DNA insert in the *DAYSLEEPER* locus [12]. These plants were grown on plate containing 12 μg/ml sulfadiazine (SUL), transferred to soil and transformed after three weeks by floral-dip transformation. Transformants were selected on medium containing 12 μg/ml sulfadiazine (SUL) and 25 μg/ml kanamycin (KM), or 15 μg/ml phosphinotrycin (PPT). SUL was added to select for the insert in the *DAYSLEEPER* locus and KM (pCAMBIA2300 based vectors) or PPT (pEARLEYGATE based vectors) to select for the complementing construct. Homo- or heterozygosity for the T-DNA insert in the *DAYSLEEPER* locus was assessed by PCR. Plants identified in the PCR screen described above were verified with RT-PCR on cDNA made from total RNA isolates. RNA was isolated from 0.1 gram of plant tissue using a Qiagen RNeasy Mini kit (Qiagen®). RNA samples were treated with DNAse (Ambion®) to get rid of residual genomic DNA. cDNA was created using an iScript cDNA synthesis kit (Biorad®). cDNA samples were diluted five times and 1 μl was used for PCR. All cDNA samples were tested by PCR, amplifying housekeeping gene ROC, using primers ROC3.3 and ROC5.2. Primers MK111 and MK112 were used to detect transcription of the native *DAYSLEEPER* locus (Additional file 1: Table S1). The amplicon in this PCR spans 235bp from the C-terminus of the *DAYSLEEPER* CDS to the 3’UTR. This PCR reaction was performed on a Biometra T1 Thermocycler (Biometra®) using a standard PCR protocol with 40 cycles (30 seconds at 95°C, 30 seconds at 59°C and 30 seconds at 72°C) with REDTaq polymerase (Sigma-Aldrich®).

### T-DNA insertion lines

Two T-DNA insertion rice lines were ordered from POSTECH; PFG_1D-01516 and PFG_1B-21919 [24]. These lines are respectively in a Daesan and Dongjin background. The first line contains a T-DNA insert in the CDS of *RICESLEEPER*1 (LOC_Os05g14940), the second line contains an insert in the 3’UTR of the *RICESLEEPER*2 (LOC_Os03g52310) gene. These lines were resistant to hygromycin and the insert in the respective loci was verified by PCR using primer combination MK85-MK101 for the *RICESLEEPER*1 gene and MK85-MK102 for the *RICESLEEPER*2 gene (Additional file 1: Table S1). To verify the presence of the wild-type loci, primers MK70-MK101 and MK102-MK105 were used, respectively. A homozygous Arabidopsis insertion line, SALK_020839C, was obtained from NASC [38]. This line has a T-DNA integrated in both alleles in the CDS of At1G15300 (*CYTOSLEEPER*).

### *Arabidopsis* protoplast transformation

*Arabidopsis thaliana* Col-0 suspension cells were used to isolate and transform protoplasts according to [39]. Protoplasts were observed after 16–18 hours of incubation at 25^O^C in the dark on a Zeiss Observer (Zeiss ®) confocal microscope. YFP was visualized using a 63x water objective and an Argon laser at 514 nm for excitation and a 522-532nm band pass emission filter. Images were processed using ImageJ (ImageJ, NIH) and Adobe Photoshop CS5 (Adobe ®).

### Transcript analysis

To analyze the 5’ UTR sequences of the *RICESLEEPER*1 and 2 gene, 1 ug of total RNA from *Oryza sativa* var. japonica seedlings was treated with DNAse (Ambion®) to remove residual genomic DNA. cDNA was created using RevertAid™ H Minus Reverse Transcriptase (Fermentas®), using oligo dT primers according to the recommended protocol. The cDNA was diluted 10x and 1 μl of this dilution was used per PCR reaction. PCR’s were performed using Phusion® polymerase in HF buffer (Finnzymes®). For cloning the 5’ noncoding leader of *RICESLEEPER*1 and 2, primers were designed to bind the first bases of the *RICESLEEPER* coding sequence (MK122 and MK125, respectively, Additional file 3: Table S2). Forward primers were designed based on EST sequences up to 1.5kb upstream of the start codon (MK120, MK121, MK123 and MK124; Figure 6 and Additional file 3: Table S2). The obtained amplicons were cloned into pJET1.2 (Fermentas®) and sequenced. All PCR’s were also performed on RNA, to test for residual gDNA in these samples. No bands were amplified from RNA samples.

### Graphics creation

Figures were created in Microsoft Office Powerpoint 2010 (Microsoft®) and edited in Adobe Photoshop CS5 (Adobe®). Visualization of conserved *SLEEPER* sequences was performed with the WebLogo on-line service [40].

## Authors’ contributions

MK performed the experiments and data processing in this study. SdP supervised the experiments and preparation of the manuscript. All authors have contributed to the study design. PJJH coordinated and helped to draft the manuscript. All authors have read and approved the final manuscript.

## Supplementary Material

Additional file 1 **Table S1.** Sequences found using TBLASTN queries in EST databases of *Amborella trichopoda* (AAGP) and mixed conifer libraries (TIGR) [18] and BLASTN in the Phytozome [19]*Selaginella* genomic database. This table was created using the DAYSLEEPER amino acid sequence as a query (TBLASTN) and the DNA coding sequence of *DAYSLEEPER* (BLASTN). The top three of Amborella hits, the three conifer hits and three top Selaginella hits are displayed, including the sequence identifier, species name (conifers) and the BLAST-scores and E-values. Standard BLAST parameters were used for TBLASTN queries, for BLASTN queries the expect threshold was increased to 100. Click here for file

Additional file 2 **Figure S1.** Synteny between the pericentromeric region of chromosome 3 of *Arabidopsis thaliana* and chromosome 11 of *Vitis vinifera.* The genes (1–5) depicted were also used in a comparison between *Brassicaceae* species by Hall et al. [22]. Gene 3 of the grapevine genome represents *VINESLEEPER2.* “CEN” is the centromere. Click here for file

Additional file 3 **Table S2.** Primerlist. Primer names, descriptions and sequences are shown. Click here for file

Additional file 4 **Figure S2.** Phylogenetic tree of *Arabidopsis thaliana* and *Capsella rubella SLEEPERS*. We estimated the non-synonymous substitution rate (Ka), synonymous substitution rate (Ks) and Ka/Ks values between DAYSLEEPER and CYTOSLEEPER and their respective homologs in *Capsella rubella.* We used the coding sequences of the genes that we found using BLASTN with the DAYLEEPER and CYTOSLEEPER coding sequences in the Phytozome [19]*Capsella rubella* genomic database. We found hits for both queries with scores of 2786 and 1891 respectively both with an E-value of 0. A FASTA file containing the unaligned coding sequences were used in the online “Ka/Ks Calculation tool” of the Bergen Center for Computational Science (http://services.cbu.uib.no/tools/kaks) [41,42]. The values in the phylogenetic tree represent the calculated Ka/Ks ratio’s. Click here for file

Additional file 5 **Figure S3.** Model of the domestication of a hAT transposase by a retrocopy process. This figure is based on a figure by Vaknin et al. [43]. An active hAT transposase gene is transcribed into mRNA. A promiscuous reverse-transcriptase reverse-transcribes the spliced mRNA into cDNA, which subsequently becomes inserted in the genome. This process results in a retrocopy that is devoid of any introns and regulatory sequences. Promoter and UTR sequences can be obtained by the retrocopy from its neighboring sequences, or by a nearby secondary integration event of another transposable element. Acquisition of UTR’s or coding material is a process called exonisation and can eventually yield a functional and actively transcribed gene: a retrogene. Overlapping ellipses represent hAT transposon associated host duplications and the adjacent arrows represent terminal inverted repeats. UTR’s are depicted as light grey boxes, coding sequences as dark grey boxes and promoter boxes as white boxes. Exons are indicated with lines. Start of transcription is marked by an arrow. Click here for file

Additional file 6 **Table S3.** Plasmids used for localization of SLEEPER fusion proteins in protoplasts and complementation of the *daysleeper* phenotype in *Arabidopsis thaliana.* Collection number and brief description and purpose in this work are shown. Click here for file

## References

[B1] Jurka J Kapitonov V Kohany O Jurka M Repetitive sequences in complex genomes: structure and evolution Annu Rev Genomics Hum Genet 2007 8 241 259 10.1146/annurev.genom.8.080706.092416 17506661

[B2] Lander ES Linton LM Birren B Initial sequencing and analysis of the human genome Nature 2001 409 860 921 10.1038/35057062 11237011

[B3] Schnable PS Ware D Fulton RS The B73 maize genome: complexity, diversity, and dynamics Science 2009 326 1112 1115 10.1126/science.1178534 19965430

[B4] Faulkner GJ Carninci P Altruistic functions for selfish DNA Cell Cycle 2009 8 2895 2900 10.4161/cc.8.18.9536 19736519

[B5] Feschotte C Transposable elements and the evolution of regulatory networks Nat Rev Genet 2008 9 397 405 10.1038/nrg2337 18368054 PMC2596197

[B6] Feschotte C Pritham EJ DNA transposons and the evolution of eukaryotic genomes Annu Rev Genet 2007 41 331 368 10.1146/annurev.genet.40.110405.090448 18076328 PMC2167627

[B7] Aravind L The BED finger, a novel DNA-binding domain in chromatin-boundary-element-binding proteins and transposases Trends Biochem Sci 2000 25 421 423 10.1016/S0968-0004(00)01620-0 10973053

[B8] Sinzelle L Izsvák Z Ivics Z Molecular domestication of transposable elements: from detrimental parasites to useful host genes Cell Mol Life Sci 2009 66 1073 1093 10.1007/s00018-009-8376-3 19132291 PMC11131479

[B9] Hudson ME Lisch DR Quail PH The FHY3 and FAR1 genes encode transposase-related proteins involved in regulation of gene expression by the phytochrome a-signaling pathway Plant J 2003 34 453 471 10.1046/j.1365-313X.2003.01741.x 12753585

[B10] Casola C Hucks D Feschotte C Convergent domestication of pogo-like transposases into centromere-binding proteins in fission yeast and mammals Mol Biol Evol 2008 25 29 41 17940212 10.1093/molbev/msm221 PMC2268608

[B11] Pritham EJ Transposable elements and factors influencing their success in eukaryotes J Hered 2009 100 648 655 10.1093/jhered/esp065 19666747 PMC2877548

[B12] Bundock P Hooykaas P An Arabidopsis hAT-like transposase is essential for plant development Nature 2005 436 282 284 10.1038/nature03667 16015335

[B13] Yamashita D Komori H Higuchi Y Yamaguchi T Osumi T Hirose F Human DNA replication-related element binding factor (hDREF) self-association via hATC domain is necessary for its nuclear accumulation and DNA binding J Biol Chem 2007 282 7563 7575 17209048 10.1074/jbc.M607180200

[B14] Benjak A Forneck A Casacuberta JM Genome-wide analysis of the “cut-and-paste” transposons of grapevine PLoS One 2008 3 e3107 10.1371/journal.pone.0003107 18769592 PMC2528002

[B15] Jiao Y Deng XW A genome-wide transcriptional activity survey of rice transposable element-related genes Genome Biol 2007 8 R28 10.1186/gb-2007-8-2-r28 17326825 PMC1852403

[B16] Rubin E Lithwick G Levy AA Structure and evolution of the hAT transposon superfamily Genetics 2001 158 949 957 11454746 10.1093/genetics/158.3.949 PMC1461711

[B17] Johnson M Zaretskaya I Raytselis Y Merezhuk Y McGinnis S Madden TL NCBI BLAST: a better web interface Nucleic Acids Res 2008 36 W5 W9 10.1093/nar/gkn201 18440982 PMC2447716

[B18] Childs KL Hamilton JP Zhu W Ly E Cheung F Wu H Rabinowicz PD Town CD Buell CR Chan AP The TIGR plant transcript assemblies database Nucleic Acids Res 2007 35 D846 D851 10.1093/nar/gkl785 17088284 PMC1669722

[B19] Goodstein DM Shu S Howson R Neupane R Hayes RD Fazo J Mitros T Dirks W Hellsten U Putnam N Rokhsar DS Phytozome: a comparative platform for green plant genomics Nucleic Acids Res 2012 40 D1178 D1186 10.1093/nar/gkr944 22110026 PMC3245001

[B20] Dong Q Lawrence CJ Schlueter SD Wilkerson MD Kurtz S Lushbough C Brendel V Comparative plant genomics resources at PlantGDB Plant Physiol 2005 139 610 618 10.1104/pp.104.059212 16219921 PMC1255980

[B21] Stamatakis A Hoover P Rougemont J A rapid bootstrap algorithm for the RAxML Web servers Syst Biol 2008 57 758 771 10.1080/10635150802429642 18853362

[B22] Hall AE Kettler GC Preuss D Dynamic evolution at pericentromeres Genome Res 2006 16 355 364 10.1101/gr.4399206 16461884 PMC1415207

[B23] Ouyang S Zhu W Hamilton J Lin H Campbell M Childs K Thibaud-Nissen F Malek RL Lee Y Zheng L Orvis J Haas B Wortman J Buell CR The TIGR rice genome annotation resource: improvements and new features Nucleic Acids Res 2007 35 D883 D887 10.1093/nar/gkl976 17145706 PMC1751532

[B24] Jeong D-H An S Park S Kang H-G Park G-G Kim S-R Sim J Kim Y-O Kim M-K Kim S-R Kim J Shin M Jung M An G Generation of a flanking sequence-tag database for activation-tagging lines in japonica rice Plant J 2006 45 123 132 10.1111/j.1365-313X.2005.02610.x 16367959

[B25] Jeon JS Lee S Jung KH Jun SH Jeong DH Lee J Kim C Jang S Yang K Nam J An K Han MJ Sung RJ Choi HS Yu JH Choi JH Cho SY Cha SS Kim SI An G T-DNA insertional mutagenesis for functional genomics in rice Plant J 2000 22 561 570 10.1046/j.1365-313x.2000.00767.x 10886776

[B26] Winter D Vinegar B Nahal H Ammar R Wilson GV Provart NJ An “electronic fluorescent pictograph” browser for exploring and analyzing large-scale biological data sets PLoS One 2007 2 e718 10.1371/journal.pone.0000718 17684564 PMC1934936

[B27] Wang W Zheng H Fan C Li J Shi J Cai Z Zhang G Liu D Zhang J Vang S Lu Z Wong GK-S Long M Wang J High rate of chimeric gene origination by retroposition in plant genomes Plant Cell 2006 18 1791 1802 10.1105/tpc.106.041905 16829590 PMC1533979

[B28] Brosius J Retroposons–seeds of evolution Science 1991 251 753 10.1126/science.1990437 1990437

[B29] Baertsch R Diekhans M Kent WJ Haussler D Brosius J Retrocopy contributions to the evolution of the human genome BMC Genomics 2008 9 466 10.1186/1471-2164-9-466 18842134 PMC2584115

[B30] Kong H Landherr LL Frohlich MW Leebens-Mack J Ma H DePamphilis CW Patterns of gene duplication in the plant SKP1 gene family in angiosperms: evidence for multiple mechanisms of rapid gene birth Plant J 2007 50 873 885 10.1111/j.1365-313X.2007.03097.x 17470057

[B31] Smith SA Beaulieu JM Donoghue MJ An uncorrelated relaxed-clock analysis suggests an earlier origin for flowering plants Proc Natl Acad Sci U S A 2010 107 5897 5902 10.1073/pnas.1001225107 20304790 PMC2851901

[B32] Stuart-Rogers C Flavell AJ The evolution of Ty1-copia group retrotransposons in gymnosperms Mol Biol Evol 2001 18 155 163 10.1093/oxfordjournals.molbev.a003789 11158374

[B33] Thompson JD Gibson TJ Higgins DG Multiple sequence alignment using ClustalW and ClustalX Curr Protoc Bioinformatics 2002 Chapter 2 Unit 2 10.1002/0471250953.bi0203s00 18792934

[B34] Waterhouse AM Procter JB Martin DMA Clamp M Barton GJ Jalview Version 2–a multiple sequence alignment editor and analysis workbench Bioinformatics 2009 25 1189 1191 10.1093/bioinformatics/btp033 19151095 PMC2672624

[B35] Earley KW Haag JR Pontes O Opper K Juehne T Song K Pikaard CS Gateway-compatible vectors for plant functional genomics and proteomics Plant J 2006 45 616 629 10.1111/j.1365-313X.2005.02617.x 16441352

[B36] Dhonukshe P Huang F Galvan-Ampudia CS Mähönen AP Kleine-Vehn J Xu J Quint A Prasad K Friml J Scheres B Offringa R Plasma membrane-bound AGC3 kinases phosphorylate PIN auxin carriers at TPRXS(N/S) motifs to direct apical PIN recycling Development 2010 137 3245 3255 10.1242/dev.052456 20823065

[B37] den Dulk-Ras A Hooykaas PJ Electroporation of agrobacterium tumefaciens Methods Mol Biol 1995 55 63 72 8528423 10.1385/0-89603-328-7:63

[B38] Scholl RL May ST Ware DH Seed and molecular resources for Arabidopsis Plant Physiol 2000 124 1477 1480 10.1104/pp.124.4.1477 11115863 PMC1539300

[B39] Schirawski J Planchais S Haenni AL An improved protocol for the preparation of protoplasts from an established Arabidopsis thaliana cell suspension culture and infection with RNA of turnip yellow mosaic tymovirus: a simple and reliable method J Virol Methods 2000 86 85 94 10.1016/S0166-0934(99)00173-1 10713379

[B40] Crooks GE Hon G Chandonia J-M Brenner SE WebLogo: a sequence logo generator Genome Res 2004 14 1188 1190 10.1101/gr.849004 15173120 PMC419797

[B41] Siltberg J Liberles DA A simple covarion-based approach to analyse nucleotide substitution rates J Evol Biol 2002 15 588 594 10.1046/j.1420-9101.2002.00416.x

[B42] Liberles DA Evaluation of methods for determination of a reconstructed history of gene sequence evolution Mol Biol Evol 2001 18 2040 2047 10.1093/oxfordjournals.molbev.a003745 11606700

[B43] Vaknin K Goren A Ast G TEs or not TEs? That is the evolutionary question. J Biol 2009 8 83 10.1186/jbiol188 PMC2776909 19863763

