# Supplementary material for: The SLEEPER genes: a transposase-derived angiosperm-specific gene family
Source: BMC Plant Biol. 2012 Oct 16;12:192. doi: 10.1186/1471-2229-12-192 (PMC3499209; doi:10.1186/1471-2229-12-192)
Supplement: Additional file 1 — Table S1. Sequences found using TBLASTN queries in EST databases of Amborella trichopoda (AAGP) and mixed conifer libraries (TIGR) [18] and BLASTN in the Phytozome [19]Selaginella genomic database. This table was created using the DAYSLEEPER amino acid sequence as a query (TBLASTN) and the DNA coding sequence of DAYSLEEPER (BLASTN). The top three of Amborella hits, the three conifer hits and three top Selaginella hits are displayed, including the sequence identifier, species name (conifers) and the BLAST-scores and E-values. Standard BLAST parameters were used for TBLASTN queries, for BLASTN queries the expect threshold was increased to 100. [file 1471-2229-12-192-S1.docx]

|  |  | **Full Length DAYSLEEPER** |  |  |
| --- | --- | --- | --- | --- |
| **TBLASTN** |  | **Sequence** | **Score** | **E-value** |
| **Amborella EST** | | b4_c10128 | 432 | 1,25E+04 |
|  |  | b4_c45898 | 300 | 5,86E+04 |
|  |  | b4_c12734 | 272 | 1,12E+04 |
| **Conifers EST** | | DV971305 (Picea glauca) | 99 | 1,50E-01 |
|  |  | TA20606_3330 (Picea glauca) | 94 | 5,30E-01 |
|  |  | ES260959 (Picea Sitchensis) | 87 | 3,50E+00 |
| **BLASTN** |  |  |  |  |
| **Selaginella moellendorffi, genomic** | | scaffold_53 | 41 | 2,05E-01 |
|  |  | scaffold_7 | 41 | 2,05E-01 |
|  |  | scaffold_120 | 39 | 7,14E-01 |
