# Supplementary material for: The SLEEPER genes: a transposase-derived angiosperm-specific gene family
Source: BMC Plant Biol. 2012 Oct 16;12:192. doi: 10.1186/1471-2229-12-192 (PMC3499209; doi:10.1186/1471-2229-12-192)
Supplement: Additional file 2 — Figure S1. Synteny between the pericentromeric region of chromosome 3 of Arabidopsis thaliana and chromosome 11 of Vitis vinifera. The genes (1–5) depicted were also used in a comparison between Brassicaceae species by Hall et al. [22]. Gene 3 of the grapevine genome represents VINESLEEPER2. “CEN” is the centromere. [file 1471-2229-12-192-S2.pdf]

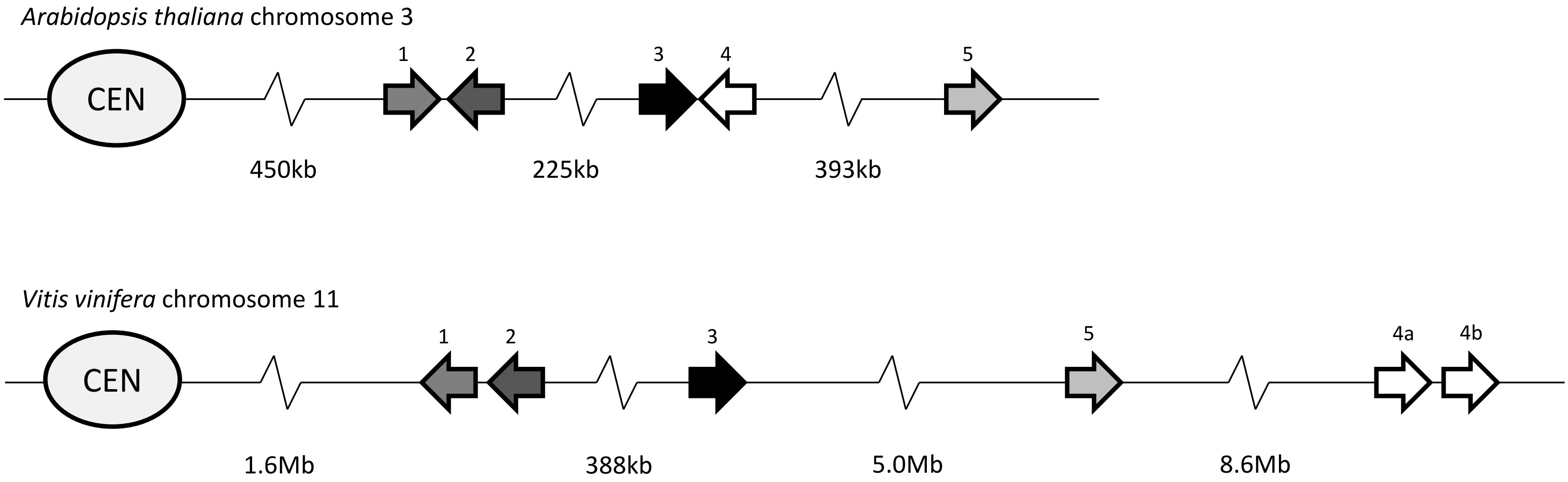

Number	Locus in *Arabidopsis*	Name
1	At3g33530	Transducin family protein
2	At3g33520	Actin-related protein 6 (ARP6)
3	At3g42170	DAYSLEEPER
4	At3g42180	Exostosin family protein
5	At3g42630	Pentatricopeptide repeat protein
