# Supplementary material for: The SLEEPER genes: a transposase-derived angiosperm-specific gene family
Source: BMC Plant Biol. 2012 Oct 16;12:192. doi: 10.1186/1471-2229-12-192 (PMC3499209; doi:10.1186/1471-2229-12-192)
Supplement: Additional file 3 — Table S2. Primerlist. Primer names, descriptions and sequences are shown. [file 1471-2229-12-192-S3.docx]

| **Primer** | **Locus** | **Sequence** |
| --- | --- | --- |
| MK3.3 | *DAYSLEEPER* promoter amplification, forward | GGTACCATGGTCTTTGCAACATAACATAAAAAGG |
| MK9.3 | *DAYSLEEPER* promoter amplification, reverse | GAGCTCTCTGCTTATTCCTTCTGA |
| MK43 | *DAYSLEEPER* promoter gateway primer, forward | GGGGACAAGTTTGTACAAAAAAGCAGGCTGAATAGAAGCAACAAAGGAAATTTTAC |
| MK44 | *DAYSLEEPER* promoter gateway primer, reverse | GGGGACCACTTTGTACAAGAAAGCTGGGTATGCTTCAGATTTGATGGTAGCA |
| MK58 | *VINESLEEPER2* reverse | GGGGACCACTTTGTACAAGAAAGCTGGGTGAAATTCCATTTTCACAAGAGCATTT |
| MK59 | *VINESLEEPER1* reverse | GGGGACCACTTTGTACAAGAAAGCTGGGTGAAATTCCATTTTCACAATTGGTGGG |
| MK60 | *DAYSLEEPER* forward | GGGGACAAGTTTGTACAAAAAAGCAGGCTCCATGGAAGTGTACAATGACGATACTG |
| MK62 | *VINESLEEPER1* forward | GGGGACAAGTTTGTACAAAAAAGCAGGCTCCATGGAGGATACCTTTACTCCCAAC |
| MK63 | *VINESLEEPER2* forward | GGGGACAAGTTTGTACAAAAAAGCAGGCTCCATGGAAACCCCTAGTGAAAACAATG |
| MK74 | *RICESLEEPER4* forward | GGGGACAAGTTTGTACAAAAAAGCAGGCTCCATGTGTGAACCAAGTGGCAGTGATG |
| MK75 | *RICESLEEPER4* reverse | GGGGACCACTTTGTACAAGAAAGCTGGGTCGTTCTCCATCTTCACCAACGCTGTG |
| MK76 | *RICESLEEPER2* forward | GGGGACAAGTTTGTACAAAAAAGCAGGCTCCATGACTGAGGAAACTGGCAACGAC |
| MK77 | *RICESLEEPER2* reverse | GGGGACCACTTTGTACAAGAAAGCTGGGTCTGGTGCATCCACCTTGACAAGCGTG |
| MK78 | *RICESLEEPER1* forward | GGGGACAAGTTTGTACAAAAAAGCAGGCTCCATGGCTGAGGAAACCAGCAACGAC |
| MK79 | *RICESLEEPER1* reverse | GGGGACCACTTTGTACAAGAAAGCTGGGTCTGCTGCATCCACCTTGACCAGCGC |
| MK80 | *RICESLEEPER3* forward | GGGGACAAGTTTGTACAAAAAAGCAGGCTCCATGGATGAGATGATACCTAAGCC |
| MK81 | *RICESLEEPER3* reverse | GGGGACCACTTTGTACAAGAAAGCTGGGTCTGGCGAATACTGGAGCCAGTCCTT |
| MK82 | *CYTOSLEEPER* forward | GGGGACAAGTTTGTACAAAAAAGCAGGCTTCATGGACACTAATCCTTCAGAGCTGG |
| MK83 | *CYTOSLEEPER* reverse | GGGGACCACTTTGTACAAGAAAGCTGGGTCTAAAGTTCCATTTTTTAACCAGTCC |
| MK98 | Gateway® cassette amplification | GGAAGCTTCGCGACAAGTTTGTACAAAAAAGCTGAAC |
| MK99 | Gateway® cassette amplification | CCAAGCTTGCATGCCTGCAG |
| MK111 | RT-PCR primers *DAYSLEEPER* expression | CCGAGGGAAATGGATGAGTA |
| MK112 | RT-PCR primers *DAYSLEEPER* expression | CAAATCACACGGTGGGTTTA |
| MK120 | *RICESLEEPER*1 UTR forward A. | GTCTTCTCTTCTCGAGCGGCTCCCG |
| MK121 | *RICESLEEPER*1 UTR forward B. | CAGGTTGGTTGGGGAATGCAGTTC |
| MK122 | *RICESLEEPER*1 ATG reverse | GTCGTTGCTGGTTTCCTCAGCCAT |
| MK123 | *RICESLEEPER*2 UTR forward A. | CTCGTCCTCCTCTCCCCTGCACCTA |
| MK124 | *RICESLEEPER*2 UTR forward B. | TCTTCTCTTCTTCCTGCCTCGCGAG |
| MK125 | *RICESLEEPER*2 ATG reverse | AGTCGTTGCCAGTTTCCTCAGTCAT |
| ROC 3.3 | RT-PCR primers ROC | CCACAGGCTTCGTCGGCTTC |
| ROC 5.3 | RT-PCR primers ROC | GAACGAACAGGCGGTGAGTC |
| MK70 | *RICESLEEPER*1 genotyping | ATGGCTGAGGAAACCAGCAACGAC |
| MK85 | Rice T-DNA insert primer | ACAAGCCGTAAGTGCAAGTG |
| MK101 | *RICESLEEPER*1 genotyping | CAGGTACTGTTCCAGTTCAG |
| MK102 | *RICESLEEPER*2 genotyping | GGACAATGATTGCTCATCAC |
| MK105 | *RICESLEEPER*2 genotyping | GGACAGAATCGCAAACAAGAAG |
