# Supplementary figures and images for: The SLEEPER genes: a transposase-derived angiosperm-specific gene family
Source: BMC Plant Biol. 2012 Oct 16;12:192. doi: 10.1186/1471-2229-12-192 (PMC3499209; doi:10.1186/1471-2229-12-192)

Ka/Ks ratio

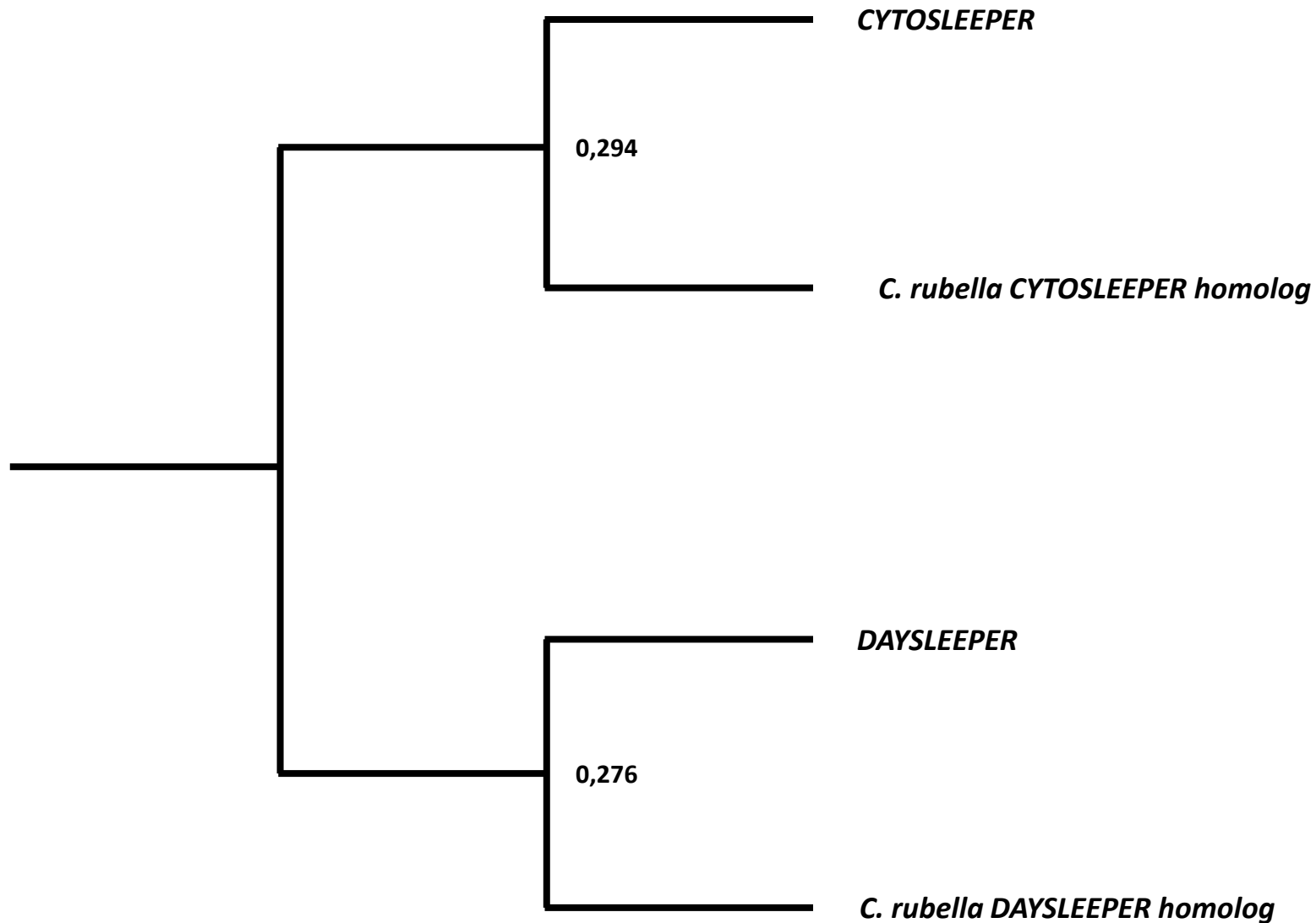

Supplement: Additional file 4 — Figure S2. Phylogenetic tree of Arabidopsis thaliana and Capsella rubella SLEEPERS. We estimated the non-synonymous substitution rate (Ka), synonymous substitution rate (Ks) and Ka/Ks values between DAYSLEEPER and CYTOSLEEPER and their respective homologs in Capsella rubella. We used the coding sequences of the genes that we found using BLASTN with the DAYLEEPER and CYTOSLEEPER coding sequences in the Phytozome [19]Capsella rubella genomic database. We found hits for both queries with scores of 2786 and 1891 respectively both with an E-value of 0. A FASTA file containing the unaligned coding sequences were used in the online “Ka/Ks Calculation tool” of the Bergen Center for Computational Science (http://services.cbu.uib.no/tools/kaks) [41,42]. The values in the phylogenetic tree represent the calculated Ka/Ks ratio’s. [file 1471-2229-12-192-S4.pdf]

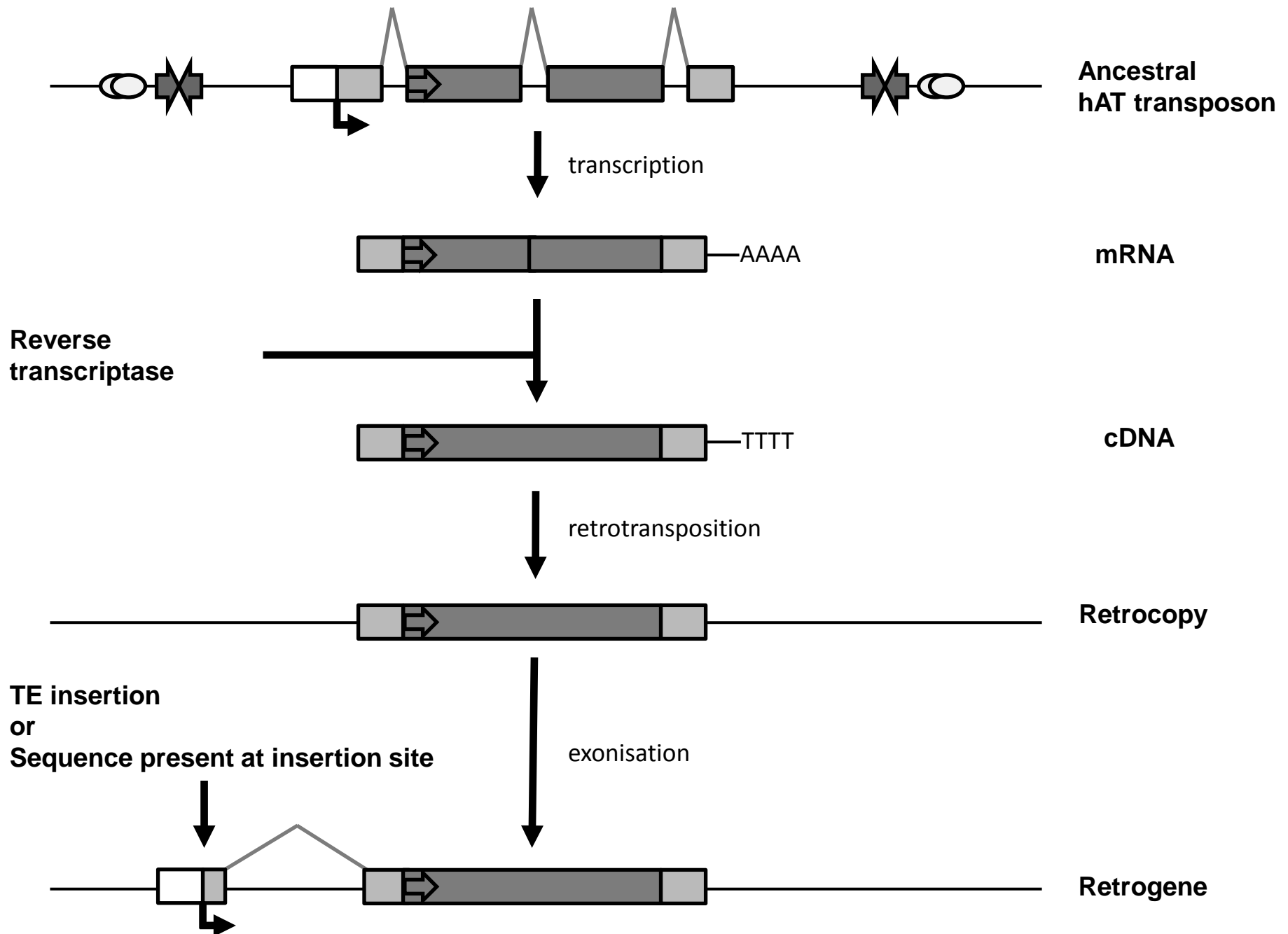

Supplement: Additional file 5 — Figure S3. Model of the domestication of a hAT transposase by a retrocopy process. This figure is based on a figure by Vaknin et al. [43]. An active hAT transposase gene is transcribed into mRNA. A promiscuous reverse-transcriptase reverse-transcribes the spliced mRNA into cDNA, which subsequently becomes inserted in the genome. This process results in a retrocopy that is devoid of any introns and regulatory sequences. Promoter and UTR sequences can be obtained by the retrocopy from its neighboring sequences, or by a nearby secondary integration event of another transposable element. Acquisition of UTR’s or coding material is a process called exonisation and can eventually yield a functional and actively transcribed gene: a retrogene. Overlapping ellipses represent hAT transposon associated host duplications and the adjacent arrows represent terminal inverted repeats. UTR’s are depicted as light grey boxes, coding sequences as dark grey boxes and promoter boxes as white boxes. Exons are indicated with lines. Start of transcription is marked by an arrow. [file 1471-2229-12-192-S5.pdf]
