# Supplementary material for: The SLEEPER genes: a transposase-derived angiosperm-specific gene family
Source: BMC Plant Biol. 2012 Oct 16;12:192. doi: 10.1186/1471-2229-12-192 (PMC3499209; doi:10.1186/1471-2229-12-192)
Supplement: Additional file 6 — Table S3. Plasmids used for localization of SLEEPER fusion proteins in protoplasts and complementation of the daysleeper phenotype in Arabidopsis thaliana. Collection number and brief description and purpose in this work are shown. [file 1471-2229-12-192-S6.docx]

| **Collection number** | **Description** | **Purpose** |
| --- | --- | --- |
| pSDM4300 | pART7 p35S Gateway YFP:HA DAYSLEEPER | Vectors used for visualizing SLEEPER cellular localization in protoplasts |
| pSDM4301 | pART7 p35S Gateway YFP:HA VINESLEEPER1 |  |
| pSDM4302 | pART7 p35S Gateway YFP:HA VINESLEEPER2 |  |
| pSDM4303 | pART7 p35S Gateway YFP:HA RICESLEEPER1 |  |
| pSDM4304 | pART7 p35S Gateway YFP:HA RICESLEEPER2 |  |
| pSDM4305 | pART7 p35S Gateway YFP:HA RICESLEEPER3 |  |
| pSDM4306 | pART7 p35S Gateway YFP:HA RICESLEEPER4 |  |
| pSDM4307 | pART7 p35S Gateway YFP:HA CYTOSLEEPER |  |
| pSDM4311 | pCAMBIA2300 pDAYSLEEPER Gateway:FLAG T_nos_ PSEUDOSLEEPER | Binary vectors used to complement the *dsl* phenotype in *Arabidopsis* |
| pSDM4312 | pCAMBIA2300 pDAYSLEEPER Gateway:FLAG T_nos_ VINESLEEPER1 |  |
| pSDM4313 | pCAMBIA2300 pDAYSLEEPER Gateway:FLAG T_nos_ VINESLEEPER2 |  |
| pSDM4314 | pCAMBIA2300 pDAYSLEEPER Gateway:FLAG T_nos_ RICESLEEPER1 |  |
| pSDM4315 | pCAMBIA2300 pDAYSLEEPER Gateway:FLAG T_nos_ RICESLEEPER2 |  |
| pSDM4316 | pCAMBIA2300 pDAYSLEEPER Gateway:FLAG T_nos_ RICESLEEPER3 |  |
| pSDM4317 | pCAMBIA2300 pDAYSLEEPER Gateway:FLAG T_nos_ RICESLEEPER4 |  |
| pSDM4318 | pEARLEYGATE302 pDAYSLEEPER::DAYSLEEPER:FLAG |  |
| pSDM4320 | pCAMBIA2300 pDAYSLEEPER Gateway :FLAG T_nos_ |  |
